# Acute Ethanol Exposure Inhibits GABA Uptake in Embryonic Chicken Retina

**DOI:** 10.1007/s11064-026-04868-7

**Published:** 2026-08-22

**Authors:** A. C. O. Damascena, A. K. Abramov, L. Pinheiro, M. Dos Santos Pereira, P. Trindade, J. Stipursky, R. A. De Melo Reis, R. C. C. Kubrusly

**Affiliations:** 1 Laboratório de Neurofarmacologia, Departamento de Fisiologia e Farmacologia, Universidade Federal Fluminense, Niterói, RJ Brazil; 2 Stanley Center for Psychiatric Research, Broad Institute of MIT and Harvard, Cambridge, MA USA; 3 Laboratório de Neuroimunologia e Desenvolvimento, Faculdade de Farmácia, Universidade Federal do Rio de Janeiro, Rio de Janeiro, RJ Brazil; 4 Laboratório de NeuroExpossoma – Biologia das Interações Neurovasculares, Instituto de Ciências Biomédicas, Universidade Federal do Rio de Janeiro, Rio de Janeiro, RJ Brazil; 5 Laboratório de Neuroquímica, Instituto de Biofísica Carlos Chagas Filho, Universidade Federal do Rio de Janeiro, Rio de Janeiro, RJ Brazil; 6 Departamento de Fisiologia e Farmacologia, UFF, Instituto Biomédico, Rua São João Batista 187, sala 416, Centro, Niterói, RJ CEP 24020-005 Brazil

**Keywords:** Ethanol, Retina, GABA, Uptake, Development

## Abstract

GABA is the major inhibitory amino acid in the central nervous system (CNS), and its homeostasis is crucial for inhibitory/excitatory balance. Its reuptake is mediated by GABA transporters, including type 1 (GAT-1), a high-affinity GABA plasma membrane transporter. Ethanol (EtOH) consumption enhances GABA inhibitory effects; however, the impact of acute EtOH exposure on GABAergic function during development remains to be understood. Therefore, we aimed to evaluate whether acute EtOH exposure modulates GABA transport in chicken retina, a well-established model for investigating neurochemical studies. GABA transporter is functional from the onset of synaptogenesis (embryonic day—E11) to E16 in chicken retina. A dose–response curve in E11 suggested that exposure to 0.1% EtOH reduced GABA uptake. This effect was not related to cell death, as cytotoxicity assays performed through lactate dehydrogenase (LDH) release showed no significant changes in E11. Additionally, inhibition of PKA and PKC pathways reversed the EtOH 0.1%-effect, suggesting the involvement of these kinases in regulating GABA transport. In contrast, Ifenprodil treatment did not alter the inhibitory effect of EtOH on GABA uptake, suggesting that GluN2B-containing NMDA receptors are not major contributors to this response. Our findings demonstrate that GABA uptake through GAT-1 during synaptogenesis may play a role in the synaptic circuitry of avian retina. Acute exposure to 0.1% EtOH did not significantly alter GABA uptake at E16 as was indicated in E11, suggesting an age-dependent effect of EtOH on retinal development, independent of cell death. These results support the hypothesis that acute EtOH exposure modulates GABA transport during retinal development, with the involvement of PKA and PKC pathways in this response.

**Supplementary Information:**

The online version contains supplementary material available at https://doi.org/10.1007/s11064-026-04868-7.

## Article Highlights

- GABA uptake is mediated by GABA transporter at the onset of chicken retina synaptogenesis (embryonic stages E11-E16).
- Exposure to 0.1% EtOH reduced GABA uptake, independent of cell death.
- Inhibition of PKA and PKC pathways reversed the EtOH 0.1%-effect.

**Supplementary Information:**

The online version contains supplementary material available at https://doi.org/10.1007/s11064-026-04868-7.

## Introduction

Ethanol (EtOH) abuse is a critical global health challenge, promoting several deaths annually and accounting for 5.3% of all global mortality [1]. An elevated alcohol abuse is found in Brazil and other American countries, reflecting a growing public health burden [2]. The abuse of EtOH among pregnant women can result in Fetal Alcohol Spectrum Disorders (FASD)—a range of irreversible conditions that include structural, cognitive, and behavioral abnormalities [3]. EtOH is able to cross the placental barrier, reaching fetal concentrations equivalent to those in maternal circulation [4, 5]. During early development, the central nervous system (CNS) is vulnerable to EtOH-induced insults disrupting neuronal differentiation, synaptogenesis, and neurotransmission [6–8].

One of the most affected neurotransmitter systems by EtOH is the GABAergic system, which is a key regulator of excitatory-inhibitory balance in the CNS [9, 10]. EtOH modulates the GABAergic system at multiple levels. It acts as a positive allosteric modulator of GABA-A receptors [8], affects GABA synthesis and receptor subunit expression [9, 10], and may alter GABA transporter (GAT) activity, GAT-1, a neuronal high-affinity GABA transporter, is responsible for the majority of GABA reuptake in the CNS and its regulation is critical for maintaining synaptic and extra synaptic GABA levels [11, 12]. Specifically in the chicken retina, over 90% of GABA uptake is GAT-1-dependent [13–16]. Pharmacological studies have established that GAT-1 activity can be selectively inhibited by compounds such as NO-711 (also referred to as NNC-711), a selective blocker of GABA uptake [17, 18]. In the chicken retina, NO-711-sensitive GABA uptake has been associated predominantly with neuronal GAT-1 activity: although both GAT-1 and GAT-3 are expressed in Müller glial cells, NO-711 does not significantly affect [^3^H]-GABA uptake in purified Müller glia cultures, where uptake is mainly mediated by GAT-3. On the other hand, NO-711 inhibits approximately 90% of [^3^H]-GABA uptake in mixed neuron–glia cultures [19, 20].

Additionally to pharmacological regulation, GAT-1 activity is modulated by PKA and PKC pathways [21], which are themselves susceptible to EtOH-mediated changes [25–27]. In addition, chronic EtOH exposure has been shown to influence glutamatergic signaling, including NMDA receptor activity. In the developing chicken retina, it enhances glutamate-evoked GABA release through NMDA receptor-dependent mechanisms [22]. Consistent with this, chronic EtOH exposure also modifies the expression of NMDA receptor subunits, particularly GluN2B [23–25]. Prenatal EtOH exposure is able to increase GluN2B-containing NMDA receptor expression and increases sensitivity to a GluN2B-selective antagonist, as ifenprodil [26], indicating functional upregulation of this subunit. Therefore, GluN2B-containing receptors seems to contribute to chronic EtOH-induced modulation of neurotransmission during development.

GABA is detectable in chicken retina at embryonic day 6 (E6) [27]. GABA is predominantly expressed in amacrine and horizontal neurons, with Müller glial cells contributing to its homeostasis via GABA transporters expression [17, 28, 29]. Remarkably, GABA uptake and release depend on GATs activity, particularly GAT-1, with evidence of physiological transporter reversal under specific ionic conditions in the chicken retina [30, 31].

Several experimental models have contributed to advancing the understanding of the effects of prenatal alcohol exposure on neurodevelopment including rodents, zebrafish, and human cerebral organoids. In rodent models, prenatal EtOH acute exposure modifies GABAergic and glutamatergic signaling, and promotes neuronal apoptosis in regions such as the hippocampus and prefrontal cortex, and modifies the expression of GABA-A receptor subunits and transporters [32, 33]. Embryonic exposure to EtOH leads to impairments in several models, as zebrafish [40], human cerebral organoids [41], rodents [18, 42, 43]. In addition, chronic gestational EtOH showed a reduction of 30% of cortical GABAergic interneurons in exposed mice Smiley et al. (2015). In other regions, like the hippocampus, prenatal EtOH similarly dysregulated GABA signaling when adult guinea pigs were exposed in utero, exhibiting elevated hippocampal GABA-A subunit expression and impairments in spatial learning [34].

Despite the contributions of these systems, the chicken retina is a powerful and accessible model for studying early neurodevelopmental processes [35]. With a highly organized laminar architecture, well-characterized developmental timeline, and a rich repertoire of neurotransmitters, it offers a tractable platform for dissecting the molecular and cellular effects of EtOH during critical windows of synaptogenesis [22, 35]. Its simplicity does not compromise its representativeness: neurotransmitter dynamics in the retina mirror those observed in central brain region [36], making it possible to investigate both GABA uptake and release, transporter regulation, and kinase involvement under precise experimental control [35].

Although chronic EtOH-induced alterations in retinal GABAergic signaling have been described [22], whether acute EtOH exposure directly modulates GABA uptake and the intracellular mechanisms involved during critical windows of synaptogenesis is largely unexplored. Given that early disruptions in GABAergic signaling have been implicated in the neurodevelopmental consequences of prenatal alcohol exposure, investigating how EtOH modulates GABA transporter function during development is crucial for advancing our understanding of neurochemical alterations associated with these effects. This study examines the effect of acute EtOH exposure on GABA uptake in the developing chicken retina, focusing on embryonic days 11 (E11) and 16 (E16) stages, characterized by active synaptogenesis and circuit refinement. By analyzing transporter activity and the involvement of signaling pathways such as PKA and PKC, this work aims to elucidate mechanisms through which acute EtOH exposure may disrupt GABAergic homeostasis during early neurodevelopment, contributing to a broader understanding of how prenatal alcohol exposure affects the developing nervous system.

## Materials and Methods

### Subjects

Fertilized White Leghorn eggs (*Gallus gallus domesticus*) were obtained from a local hatchery and staged as previously described [37]. Embryonic retinas (E11 and E16) were dissected and separated from the other ocular tissues in calcium-magnesium free solution (CMF), at 37 °C. Subsequently, retinas were placed in 35 mm petri dishes containing 1 ml of Hanks’ 4 Balanced Salt Solution (HBSS; NaCl 128 mM; KCl 4 mM; MgCl_2_ 1 mM; –CaCl_2_ 3 mM; HEPES 20 mM; glucose 4 mM; pH 7.4), for neurochemical assays.

### Drugs and Reagents

EtOH P.A. (obtained from Isofar), H-89 dihydrochloride hydrate (H-89, 10 µM; #B1427), NO-711 hydrochloride (NO-711, 100 µM; N142), Gö 6983 (2-[1-(3-dimethylaminopropyl)− 5-methoxyindol-3-yl]− 3-(1H-indol-3-yl) maleimide, 100 nM; G1918), and Ifenprodil (10 µM; I-2892) were obtained from Sigma–Aldrich (St. Louis, MO, USA). Radiolabeled GABA ([^3^H]-GABA) specific activity 35 Ci/mmol was purchased from PerkinElmer (Massachusetts, USA). LDH Cytotoxicity Assay Kit was obtained from Cayman Chemical (Ann Arbor, MI, USA). All other reagents were of analytical grade, obtained from high-grade sources.

### Tissue Preparation

Retinas from Fertilized White Leghorn eggs were isolated. To increase the contact surface, the tissue amount was equally sliced in sections of approximately 200 µm already in DMEM/F12 or Tris–HCl solution (pH 7.4) before the addition of [^3^H]-GABA (at 37 °C) or the addition of lysis buffer for western immunoblotting (20 mM Tris–HCl pH 8.0, 137 mM NaCl, 10% glycerol supplemented with protease inhibitors (10% v/v; Sigma) and phosphatase (Roche).

### EtOH and Drug Treatment

EtOH was prepared by serial dilution in HBSS solution, starting from a 1% (v/v) EtOH concentration and reaching a final concentration of 0.1% (v/v), corresponding to approximately 21.71 mM (see Table 1). In most assays, EtOH was administered for 30 min prior to the addition of [^3^H]-GABA. An exception was made for the release assay, in which EtOH was perfused for 5 min, either alone or in combination with other drugs.

**Table 1:** Drugs and concentrations used

Drug	Function	Concentration
Ethanol	Positive allosteric modulator of GABA_A_ receptors	17 mM
NO-711	GABA transporter-1 (GAT-1) inhibitor	50 µM
Gö 6983	Protein kinase C (PKC) inhibitor	100 nM
H89	Protein kinase A (PKA) inhibitor	100 nM
Ifenprodil	GluN2B-selective N-methyl-d-aspartate (NMDA) receptor antagonist	10 µM

### [^3^H]-GABA Uptake Assay

[^3^H]-GABA uptake was performed as described before [38]. After pre-exposure to NO-711 (100 μM), Gö 6983 (100 nM), H89 (10 µM), or HBSS alone (control group), each retina was incubated for 1 h in 1 mL of HBSS pH 7.4 at 37 °C containing 0.5 μCi of [^3^H]-GABA (35 Ci/mmol = 35.106 μCi) and 20 μM of non-radioactive GABA as a carrier without changing the initial medium. The GABA concentration of 20 μM and the incubation time (1 h) were selected to ensure the steady state of the uptake curve, while 0.5 μCi [3H]-GABA (35 Ci/mmol) was only used as a radioactive tracer to evaluate [3H]-GABA transport activity [18, 39]. After 60 min incubation with [^3^H]-GABA, the solution was removed, and the tissue was washout three times with 3 mL of cold HBSS. This process was sufficient to washout the radioactivity not taken up by the cells. Then, 1 mL of Milli-Q water (Milli pore) was added to disrupt cell membranes, followed by a freeze–thaw cycle. The intracellularly accumulated radioactivity was determined using a liquid scintillation counter. Uptake values were calculated as fmol of [3H]-GABA and normalized by protein concentration, estimated with Lowry protein assay using BSA as standard, as described previously [38].

### [^3^H]-GABA Release Assay

Briefly, [^3^H]-GABA release was performed as described before [38]. Following the [3H]-GABA uptake, the medium was removed, and the tissue was washed three times with 1 mL of warm HBSS at 37 °C to washout the radioactivity not taken up by the cells. Afterward, the retina was superfused with 0.5 mL of HBSS alone (basal), EtOH 0.1% (vol/vol) and/or Ifenprodil (10 µM) at 37 °C for 5 min. At the end of the superfusion period, the superfusate was collected and its radioactivity was quantified by liquid scintillation. To determine the proportion of released radioactivity relative to total cellular [3H]-GABA content, the corresponding retinal tissues were subsequently processed according to the uptake protocol. Release values were normalized as a percentage of total [^3^H]-GABA previously incorporated by each retina [40].

### Western Blot Assay

Western Blot was performed as described before [38]. Briefly, both retinas were extracted and homogenized together in a RIPA buffer containing a cocktail of protease inhibitors. The homogenate was used to assess the expression of GAT-1 protein and phosphorylated subunit GluN2B in control and EtOH-treated groups. Protein concentration was estimated [41] and the samples were diluted in a buffer composed of 10% glycerol (v/v), 1% ß-mercaptoethanol, 3% SDS and 62.5 mm Tris base, which were boiled for 5 min. Approximately 15 μg of protein from each sample was used in electrophoresis in 8% (GAT-1) and 10% (GluN2B) SDS-PAGE and transferred to polyvinylidene fluoride (PVDF) membranes (ECL-Hybond) by a semi-dry transfer method. Membranes were washed with a Tween 20 Tris-buffered saline (TTBS) and blocked for 2 h with TTBS plus 1% BSA. After blocking, membranes were lightly washed with TTBS and incubated with primary anti-GAT-1 (1:500 in TTBS; Sigma-Aldrich; HPA013341) and anti-pGluN2B antibodies (1:2000 in TTBS; Sigma-Aldrich; M2442) overnight at 4 °C. On the following day, the primary antibodies were removed, and the membranes were washed three times with TTBS to remove unconjugated antibodies. Subsequently, incubation was carried out with anti-rabbit secondary antibodies conjugated to peroxidase (1:5000 in TTBS; Sigma-Aldrich; F1262) for 2 h at room temperature. After incubation, the membrane was again washed three times with TTBS (10 min each), and the probe was detected using an ECL kit (Amersham).

Membranes were re-probed with mouse anti-β-tubulin antibody (1:25,000 in TTBS, Cell Signaling; T5201) or mouse anti-β-actin antibody (1:2000 in TTBS; #CBL171, Millipore) for 1 h at room temperature, washed 3 times with TTBS, and incubated with a secondary anti-mouse antibody conjugated to peroxidase (1:5000 in TTBS; Santa Cruz Biotechnology; SC533657) for 45 min at room temperature, followed by three new TTBS washes. Immunostaining was detected with the ECL kit. The intensity of the bands was analyzed using ImageLab 5.2.1 software (Bio-Rad Laboratories Inc).

### Lactate Dehydrogenase (LDH) Cytotoxicity Assay

Cytotoxicity was assessed by measuring lactate dehydrogenase (LDH) release, which reflects plasma membrane disruption and loss of cellular integrity [42]. The assay was performed as previously described, with adaptations for the ex vivo chick retina preparation [42]. Briefly, the LDH Cytotoxicity Assay Kit (Cayman Chemical) was used according to the manufacturer's instructions. E11 chick retinas were incubated with 0.1% EtOH or vehicle for 30 min at 37 °C, while 10% Triton X-100 was used as a positive control for maximal LDH release. After treatment, the tissues were transferred to microcentrifuge tubes, vortexed and homogenized to promote tissue dissociation. Following a brief centrifugation, 10 μL of the supernatant from each sample was transferred to a 96-well plate containing HBSS and incubated with the LDH reaction solution, freshly prepared according to the manufacturer's instructions, for 30 min at 37 °C. Absorbance was measured at 490 nm using a microplate reader. Cytotoxicity was expressed as the percentage of maximal LDH release, calculated from spontaneous and maximum LDH release controls.

### Statistical Analysis

All statistical analyses were conducted using GraphPad Prism 10 (GraphPad Software, LLC). Data are presented as mean ± standard error of the mean (SEM) (or as percentage of control where applicable), and results were considered significant at p < 0.05. Potential outliers were assessed using the ROUT (Robust Regression and Outlier Removal) method with Q = 1% in GraphPad Prism. Only values objectively identified by this criterion were excluded from the statistical analyses. For comparisons between two groups, unpaired two-tailed t-tests were used, with Welch’s correction applied whenever variances were unequal or group sizes were unbalanced. For comparisons involving three or more groups, one-way analysis of variance (ANOVA) was performed (using Welch’s ANOVA when heteroscedasticity warranted), and factorial experiments with a 2×2 design were analyzed by two-way ANOVA including the interaction term. Following Welch’s one-way ANOVA, Dunnett’s T3 post hoc test was used when the experimental design required comparisons of each treatment group with a single reference group, as this procedure appropriately accounts for unequal variances and unbalanced sample sizes. For two-way ANOVA, group differences were further examined using planned post hoc comparisons (simple effects contrasts) rather than exhaustive pairwise testing, with Šídák’s method employed to adjust for multiple comparisons, as it appropriately controls the family-wise error rate while maintaining greater statistical power for a limited number of predefined pairwise comparisons. Tukey's multiple comparisons test was used to evaluate all possible pairwise comparisons among the experimental groups while controlling the family-wise error rate. The sample size (n) refers to the number of retinas per experimental group, and retinas were randomly assigned to treatment groups.

Assumptions of normality and homogeneity of variances were verified primarily by visual inspection of residuals and Q–Q plots. Formal tests of normality (e.g., Shapiro–Wilk) were performed (Supplementary data 1) but not relied upon in isolation, given their limited sensitivity with small sample sizes. In comparisons involving small group sizes (n ≤ 4), formal statistical testing was still performed as specified above; however, these results are interpreted cautiously and are presented as exploratory and hypothesis-generating, given the limited precision and generalizability associated with small samples. No logarithmic or other data transformations were applied; all analyses were conducted on raw (untransformed) data.

## Results

### Developmental Dynamics of GABA Uptake and Its Dose‑Dependent Inhibition by Acute EtOH in the Embryonic Chicken Retina

**Fig. 1:**
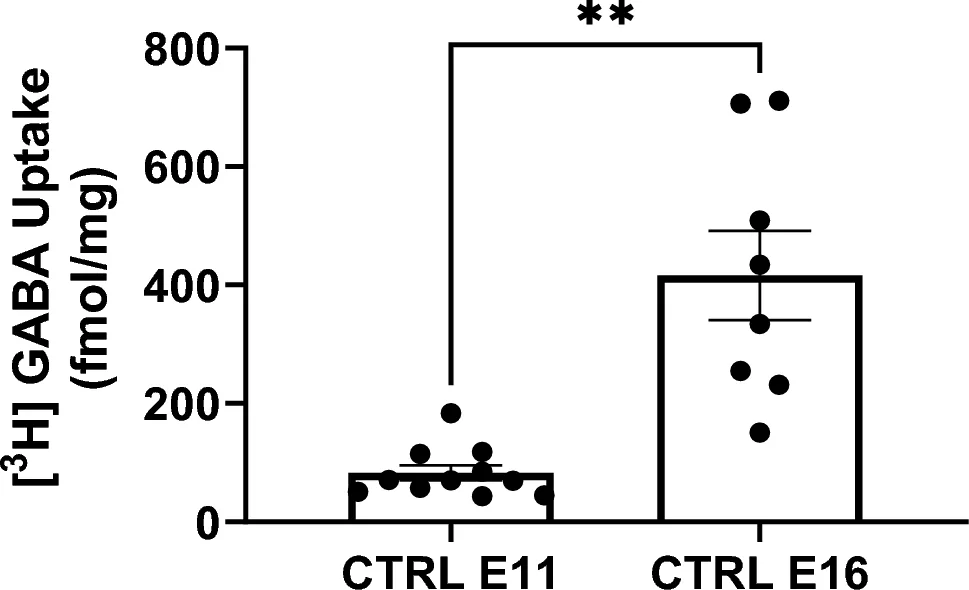
GABA uptake at different developmental stages in embryonic chicken retinas. Retinal tissues were collected at embryonic day 11 (E11) and 16 (E16) and incubated with [3H]-GABA to assess uptake activity. GABA uptake is present at E11, indicating early functional expression of GABA transporters. A significant increase in uptake was observed at E16, reflecting enhanced transporter activity during synaptic maturation. Data are expressed as mean ± SEM (n = 8–11). Statistical analysis was performed using an unpaired Welch’s t-test (i.e., not assuming equal variances). Different colors for individual dots correspond to different experiment batches. **p < 0.01

Although the retina is a well-established model for studying neurotransmission and neurodevelopmental processes, few studies have explored its neurochemical dynamics in the context of EtOH exposure, particularly regarding GABA transport mechanisms. The embryonic chicken retina offers a unique opportunity to investigate these questions due to its well-characterized developmental timeline and accessibility. However, the temporal regulation of high-affinity GABA uptake, as well as its modulation by acute EtOH exposure, remains poorly understood in this system. To establish a baseline for GABA transporter function during key neurodevelopmental windows, two distinct embryonic stages were used: embryonic day 11 (E11), corresponding to the onset of synaptogenesis and early neural circuit formation, and embryonic day 16 (E16), a stage of retinal development with active synaptic refinement [28]. [^3^H]-GABA uptake assays were conducted in retinal explants collected at E11 and E16 (Fig. 1). GABA uptake was then evaluated using [^3^H]-GABA assays, and values were normalized to protein concentration (fmol/mg). At E11, we observed measurable GABA uptake (82.7 ± 12.63 fmol/mg), indicating that GABA transporters are functionally active at the beginning of synaptic development (Fig. 1). By E16, uptake values increased significantly to 416.6 ± 75.25 fmol/mg, suggesting an upregulation of transporter activity as the retina matures and synaptic connectivity becomes more complex (p = 0.0029 vs. E16) (Fig. 1).

**Fig. 2:**
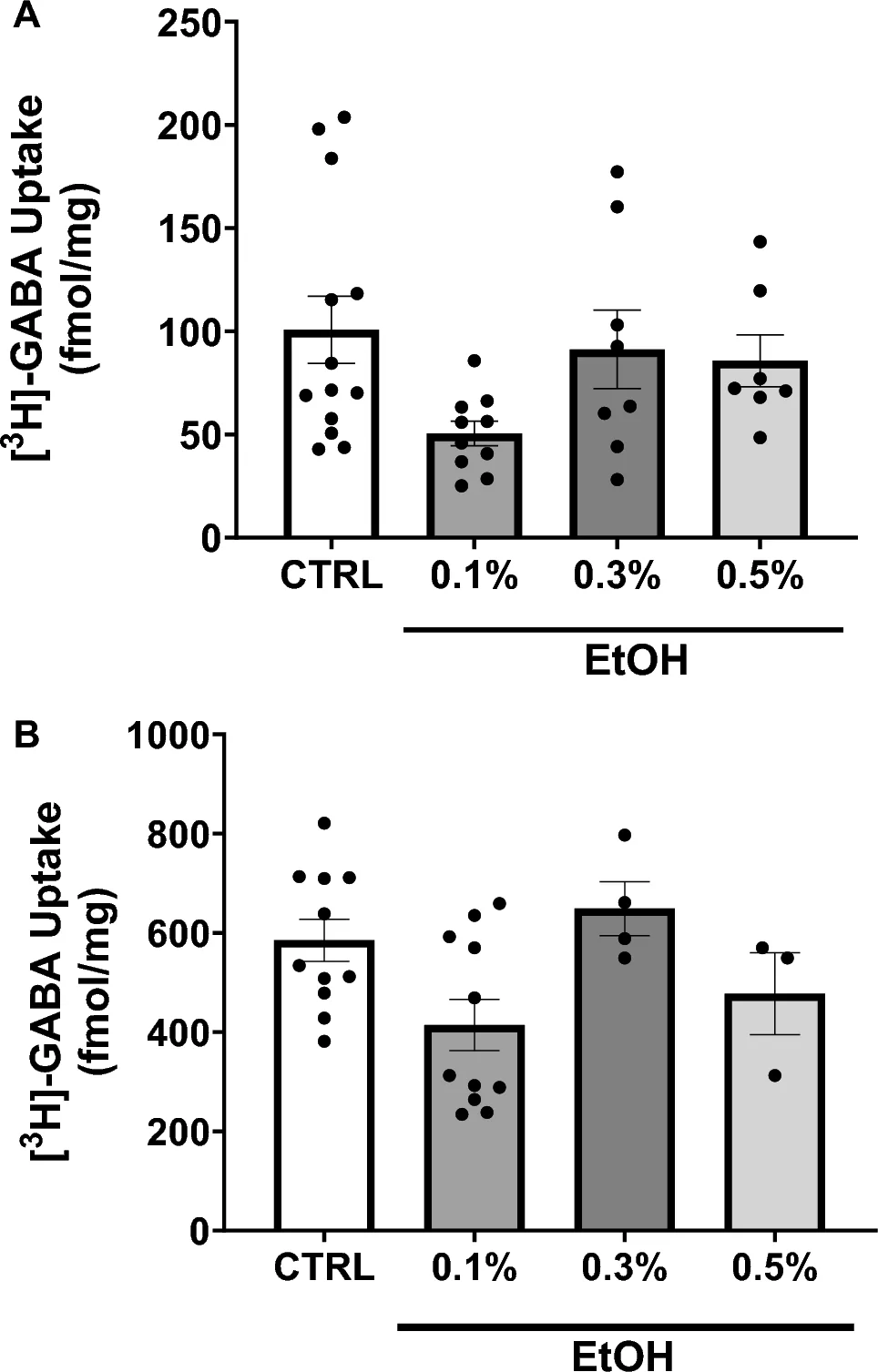
Dose-dependent effect of acute EtOH exposure on GABA uptake in E11 and E16 chicken retinas. **A** Retinal explants from E11 embryos were treated for 30 min with vehicle (CTRL) or increasing concentrations of ethanol (EtOH) (EtOH: 0.1%, 0.3% and 0.5%; v/v), and GABA uptake was measured using [3H]-GABA (n = 7–13). Acute treatment with 0.1% EtOH produced a suggestive reduction in GABA uptake compared with CTRL (p = 0.0946), whereas 0.3% and 0.5% EtOH did not differ from CTRL **B** A similar assay was conducted using explants from E16 embryos. Acute EtOH treatment did not significantly affect GABA uptake at this developmental stage. Results were normalized to protein content (fmol/mg). Data are presented as mean ± SEM. Sample sizes varied between groups (*n* = 3–11). Statistical analysis was performed using Welch’s one-way ANOVA, followed by Dunnett’s T3 post hoc test to compare each treatment group to CTRL. This approach was chosen to account for unequal variances and unbalanced sample sizes. *p < 0.05

Once GABA uptake is functional at embryonic day 11 (E11), we asked whether EtOH could modulate this process. E11 retinal explants were incubated for 30 min in HBSS containing increasing concentrations of EtOH: 0.1%, 0.3% and 0.5% (v/v). Untreated retinas incubated in HBSS alone served as the control group. Welch’s ANOVA test indicated a significant effect for EtOH treatment in E11 (W_4, 14.26_ = 7.093; p = 0.0023). As shown in Fig. 2A for E11, only the lowest concentration (0.1%) showed a suggestive decrease of GABA uptake (p = 0.0946 vs. CTRL), whereas higher doses did not differ from control (p > 0.05). At E16, EtOH treatment also did not significantly affect GABA uptake at 0.1% (p = 0.1607 vs. CTRL) (Fig. 2B). Together, these results suggest a non-linear dose effect. Importantly, the consistent tendency observed at E11 supports the selection of this developmental stage and the 0.1% EtOH concentration for subsequent experiments.

### GAT‑1 Inhibition Decreases GABA Uptake with no Change in Transporter Expression

**Fig. 3:**
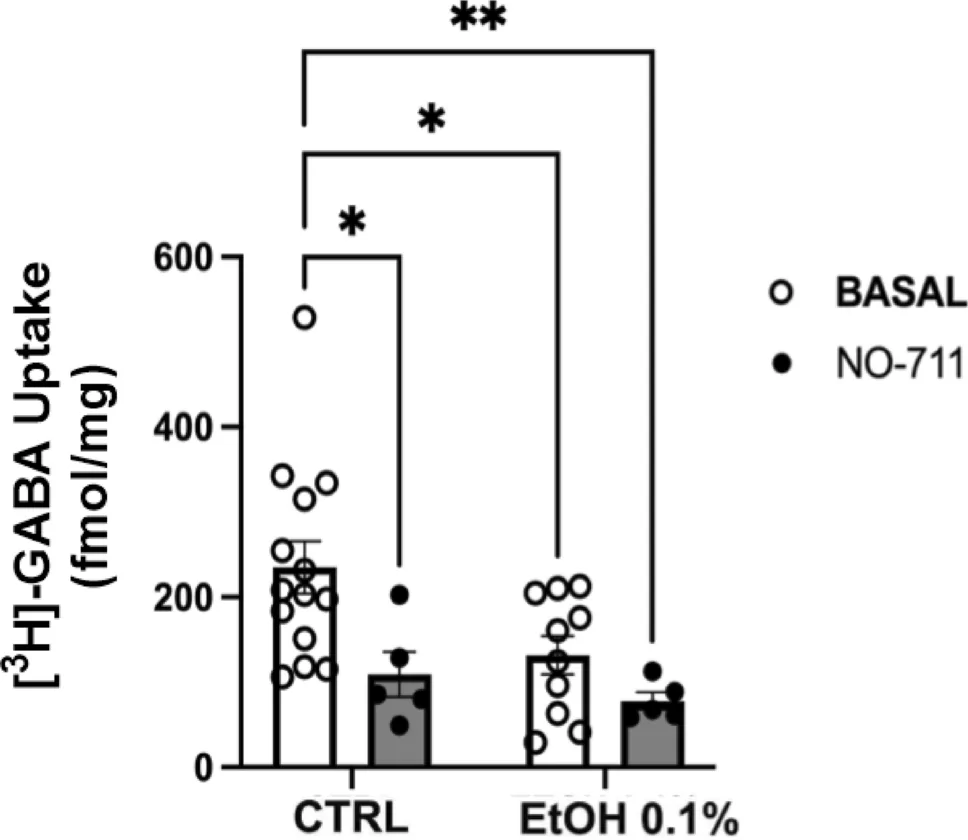
Effect of GAT-1 inhibition and EtOH on GABA uptake in E11 chicken retinas. Retinal explants were treated with vehicle (CTRL), 0.1% ethanol (EtOH), 100 µM NO-711 (GAT-1 inhibitor), or 0.1% EtOH + NO-711 for 30 min. [^3^H]-GABA uptake was measured and normalized to protein content (fmol/mg). Both EtOH and NO-711 significantly reduced GABA uptake compared to CTRL (CTRL: 228 ± 37; EtOH: 123 ± 20; NO-711: 102 ± 20 fmol/mg, *p* < 0.05). Co-treatment with EtOH + NO-711 resulted in similar uptake levels (79 ± 15 fmol/mg) with no additive effect. Data are presented as mean ± SEM (n = 5–14). Statistical analysis was performed using ANOVA followed by Tukey’s post hoc test; *p* < 0.05 vs. CTRL. *p < 0.05 and **p < 0.01

To evaluate whether the suggestive effect in GABA uptake observed following acute EtOH exposure involves GAT-1 activity, E11 retinal explants were incubated for 30 min in four groups: vehicle control (HBSS), 0.1% EtOH (EtOH), 100 µM NO-711 (a selective GAT-1 inhibitor), and the combination of 0.1% EtOH + NO-711 (Fig. 3). Treatment with NO-711 reduced [^3^H]-GABA uptake levels compared to control (CTRL: 228 ± 37; NO-711: 102 ± 22 fmol/mg, *p* < 0.05) (Fig. 3). Similarly, retinas exposed to 0.1% EtOH showed a reduced uptake (123 ± 20 fmol/mg, *p* < 0.05). The group receiving the combined treatment (EtOH + NO-711) was similar (78 ± 15 fmol/mg) with no additional reduction beyond the levels observed in the single-treatment groups (Fig. 3). These data confirm that both NO-711 and 0.1% EtOH decrease GABA uptake at E11. The absence of a more pronounced effect in the co-treated group indicates that the EtOH and GAT-1 selective inhibitor does not produce additive effects in GABA uptake pattern.

**Fig. 4:**
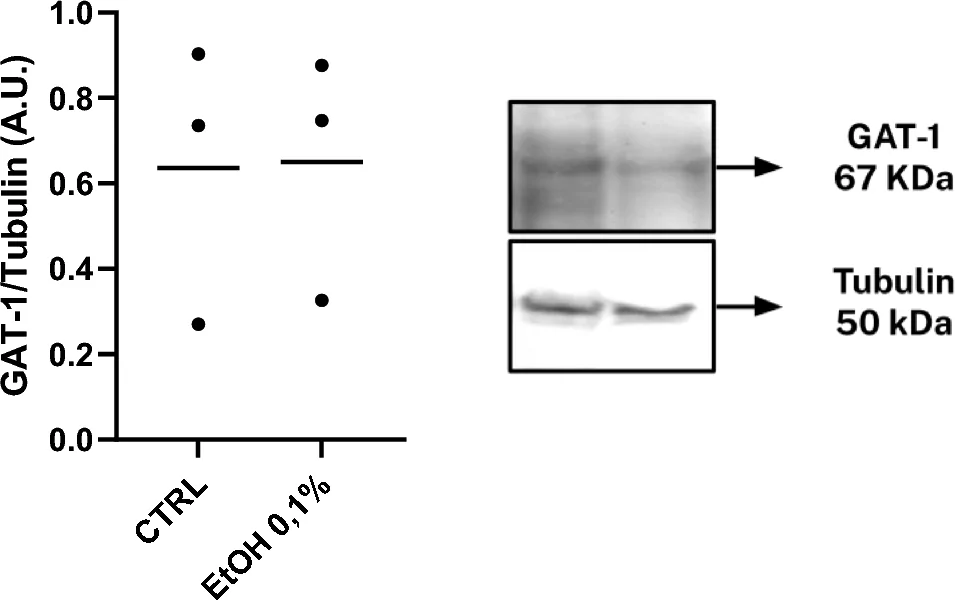
Western blot analysis of GAT-1 expression in E11 chicken retinas after acute EtOH exposure. Retinal explants were incubated with vehicle (CTRL) or 0.1% ethanol (EtOH) for 30 min. GAT-1 protein levels were measured by Western blot and normalized to β-actin. No significant difference was observed between groups (*p* = 0.9592; *n* = 3 per group). Data are expressed as mean ± SEM. Statistical analysis was performed using unpaired Welch’s t-test, which accounts for unequal variances. Given the small sample size, these results are interpreted in an exploratory, hypothesis-generating context

We next sought to determine whether EtOH modulates GABA uptake by altering GAT-1 protein expression levels. Thus, we performed Western blot analysis to assess the protein content of GAT-1 in control and EtOH-treated retinas (Fig. 4). Retinal tissues were collected from E11 embryos incubated for 30 min in either vehicle (HBSS) or 0.1% EtOH (EtOH), followed by protein extraction and immunoblotting for GAT-1. Densitometric analysis revealed no significant difference in GAT-1 protein levels between control and EtOH-treated groups (t = 0.054, df = 3.934; p = 0.9592) (Fig. 4). This data suggests that EtOH-induced decrease in GABA uptake in E11 retinas is independent of GAT-1 protein levels modulation.

### Reversible EtOH‑Induced Inhibition of GABA Uptake Occurs Without Cytotoxicity in E11 Retinas

After demonstrating that acute exposure to 0.1% EtOH significantly reduces GABA uptake in E11 retinas, and that this effect is not accompanied by changes in GAT-1 protein expression, we next investigated whether the observed functional inhibition is reversible. This step is important to determine whether EtOH transiently modulates transporter activity or induces longer-lasting disruptions in inhibitory neurotransmission during early retinal development.

**Fig. 5:**
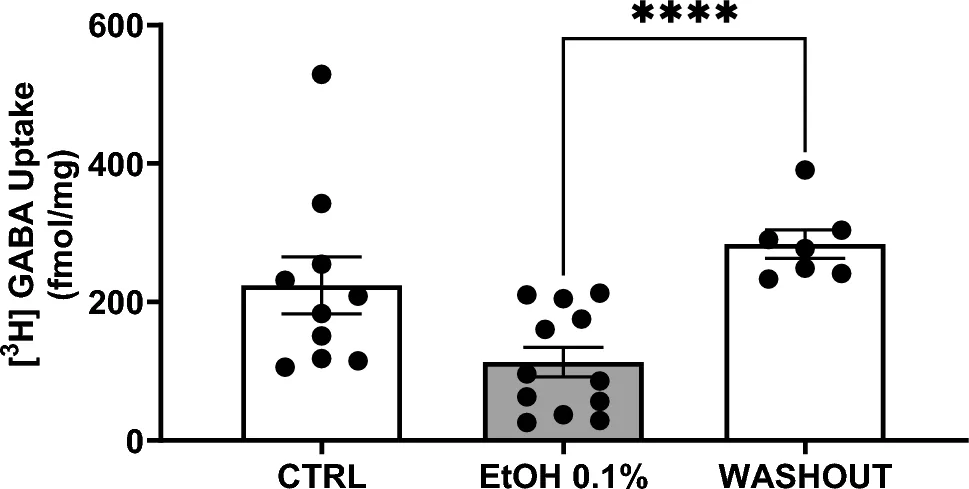
Reversibility of EtOH-induced inhibition of GABA uptake following washout. E11 chicken retinal explants were divided into three experimental groups: control (CTRL), ethanol (EtOH; 30-min exposure to 0.1% EtOH), and washout (30-min exposure to 0.1% EtOH followed by a 10-min wash with Hank’s solution 4). GABA uptake in the EtOH group showed a suggestive reduction compared to the control group (p = 0.08). Following washout, uptake was significantly restored p < 0.0001 vs. EtOH 0.1%). Data are presented as mean ± SEM (n = 7–12). Statistical analysis was performed using one-way Welch’s ANOVA followed by Dunnett T3 post hoc test, appropriate for unbalanced groups with unequal variances. Different colors for individual dots correspond to different experiment batches. *p < 0.05 and **** p < 0.0001

To assess the reversibility of EtOH’s effect, retinal explants from E11 chick embryos were exposed to 0.1% EtOH in HBSS for 30 min (Fig. 5). Next, explants were either immediately processed for [3H]-GABA uptake (EtOH group) or transferred to EtOH-free HBSS for a 10-min washout period before uptake GABA assessment (Washout group). A control group (CTRL) was incubated only in HBSS. Welch’s ANOVA pointed to statistical significance among groups (W_2, 14.10_ = 16.13; p = 0.0001). As shown in Fig. 5, EtOH pointed to a suggestive decrease GABA uptake compared to the control (p = 0.088 vs. CTRL). Remarkably, explants subjected to the washout protocol showed uptake levels that were fully restored to baseline (p = 0.5286 vs. CTRL; p < 0.0001 vs. EtOH 0.1%), indicating that the inhibitory effect of EtOH on GABA uptake is reversible under the conditions tested.

**Fig. 6:**
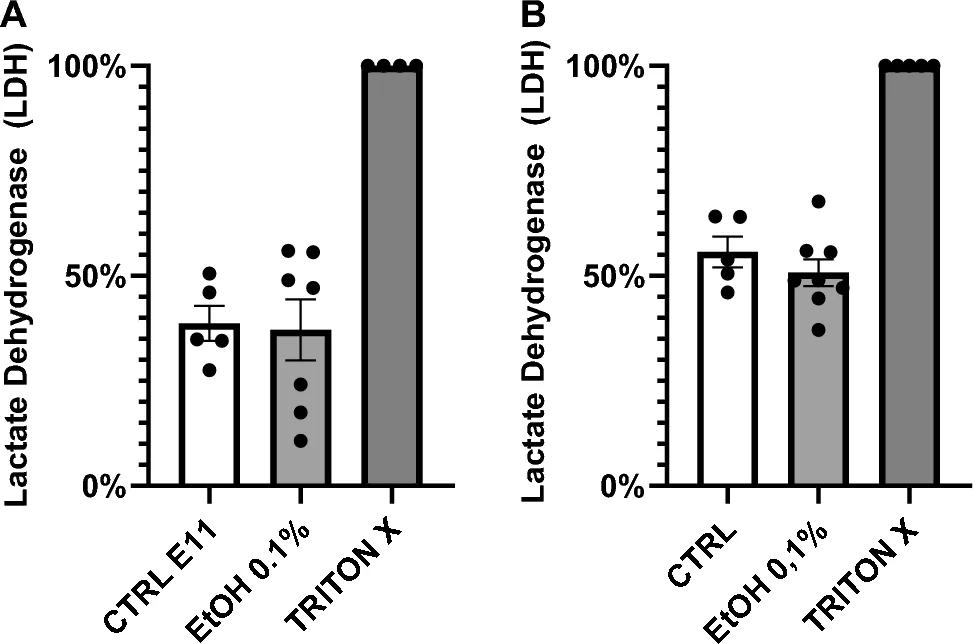
EtOH exposure does not reduce cell viability in E11 and E16 chicken retinas. Cell death was assessed by measuring lactate dehydrogenase (LDH) release in retinal explants at embryonic day 11 (E11, A) and day 16 (E16, B) following a 30-min incubation with vehicle (CTRL) or 0.1% ethanol (EtOH). A third group treated with 0.01% Triton X-100 was included as a positive control for maximal lysis. LDH release values were normalized to the Triton X group (100%) to express results as percentage of total lysis. Both CTRL and EtOH groups showed comparable LDH levels (p > 0.05), indicating no EtOH-induced increase in cell death. Triton X-100 treatment resulted in complete cell lysis, validating the assay. Statistical comparisons were performed between CTRL and EtOH groups only, using Welch’s t-test (n = 5–7 per group). The Triton group was excluded from inferential analysis and used solely as a normalization reference. Values are presented as mean ± SEM. Different colors for individual dots correspond to different experiment batches.

To demonstrate that EtOH is not cytotoxic, we assessed cell viability through quantification of lactate dehydrogenase (LDH) release, a widely used marker of membrane integrity and cell lysis. Elevated levels of LDH in the extracellular medium are indicative of cellular damage or death. As shown in Fig. 6, LDH levels in the EtOH-treated group were statistically similar to those in the control group in both ages (E11: t = 0.1913, df = 9.153; p = 0.8525; E16: t = 1.019, df = 9.437; p = 0.3335), indicating that acute exposure to 0.1% EtOH did not increase membrane permeability or induce cell death under these conditions. Triton-X addition to retinas were used as positive control and reference levels for cytotoxicity.

### EtOH Does Not Modify GluN2B Phosphorylation but Promotes GABA Release in E11 Retinas

**Fig. 7:**
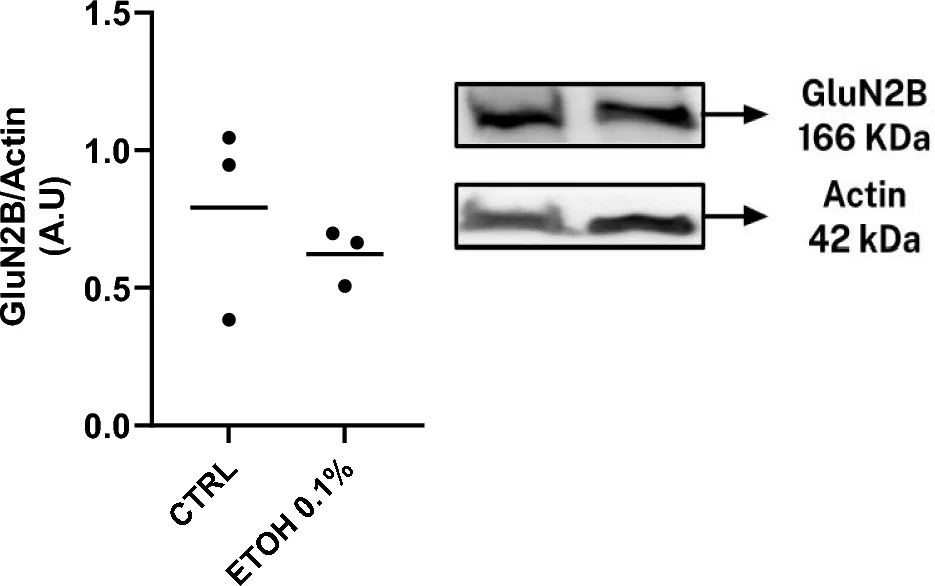
EtOH does not alter phosphorylated GluN2B expression in E11 chicken retinas. Western blot analysis of phosphorylated GluN2B (pGluN2B) was performed in retinal explants from E11 embryos treated for 30 min with vehicle (CTRL) or 0.1% ethanol (EtOH). Densitometric quantification normalized to actin revealed no significant difference between groups (n = 3 per group; p = 0.5025). Data are presented as mean ± SEM. Statistical analysis was performed using Welch’s t-test. Given the small sample size, results are interpreted as preliminary and hypothesis-generating

We asked if EtOH may influence intracellular signaling pathways that regulate transporter function. To do so, we examined the levels of phosphorylated GluN2B (pGluN2B) in retinal explants from E11 embryos following acute exposure to 0.1% EtOH for 30 min (Fig. 7). As shown, no significant difference in pGluN2B expression was observed between EtOH-treated retinas and controls (t = 0.7889, df = 2.328; p = 0.5025). These results suggest that, under the experimental conditions tested, acute exposure to low-dose EtOH does not significantly alter the phosphorylation state of the GluN2B subunit in the developing chicken retina.

**Fig. 8:**
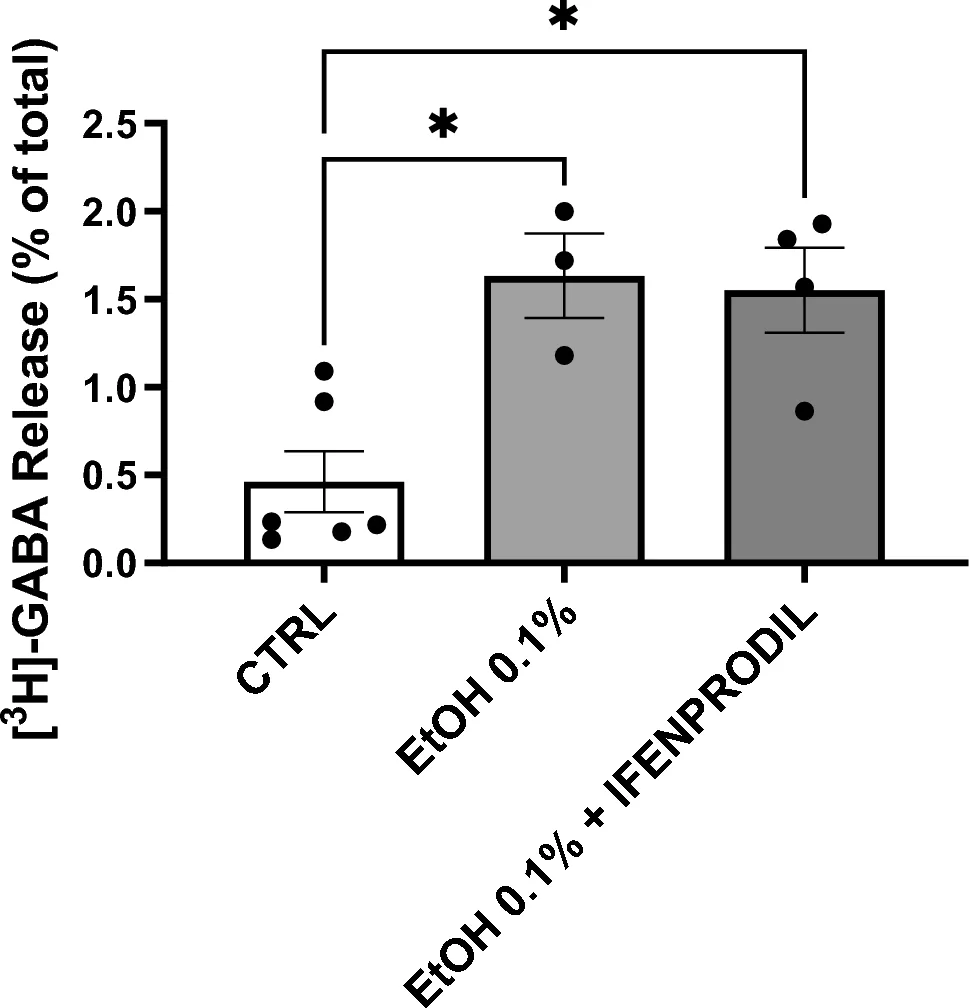
The effect of EtOH on GABA release is not inhibited by Ifenprodil. Acute exposure to 0.1% ethanol (EtOH) for 5 min significantly increased [^3^H]-GABA release in E11 chicken retinal explants compared to control. Co-application of Ifenprodil (10 µM), a selective allosteric inhibitor of GluN1-GluN2B NMDA receptors, did not reverse GABA release to baseline levels. Data are expressed as mean ± SEM (n = 3–6). Statistical analysis was performed using Welch’s ANOVA followed by Dunnett’s T3 post hoc test. p = 0.0421 vs. CTRL (EtOH 0.1%); p = 0.0285 vs. CTRL (EtOH + Ifenprodil). Given the modest sample size and variation, results are interpreted as preliminary and hypothesis-generating. Different colors for individual dots correspond to different experiment batches. *p < 0.05

Next, we asked whether acute EtOH exposure also modulates neurotransmitter release. To address this, E11 retinal explants were pulse-labeled with [^3^H]-GABA and exposed for 5 min to 0.1% EtOH, a time window selected consistent with pulse-based release paradigms in the chicken retina in previous results [17, 18, 22] (Fig. 8). A general effect was found in Welch’s ANOVA test (W_2, 5.18_ = 9.592; p = 0.0181). As shown in Fig. 8, EtOH increased GABA release, more than doubling the amount released relative to vehicle controls (p = 0.0421 vs. CTRL). To determine whether this effect involves NMDA receptors containing the GluN2B subunit, explants were co-treated with Ifenprodil (10 µM), a GluN2B antagonist. Co-application of Ifenprodil did not restore release to baseline, and GABA efflux remained elevated (p = 0.0285 vs CTRL; p = 0.9921 vs. EtOH 0.1%). These findings demonstrate that the EtOH-induced enhancement of GABA release in E11 retinas is robust and does not depend on activation of GluN2B-type NMDA receptors.

### Inhibition of PKA and PKC Reverses EtOH-Induced Reduction in GABA Uptake

**Fig. 9:**
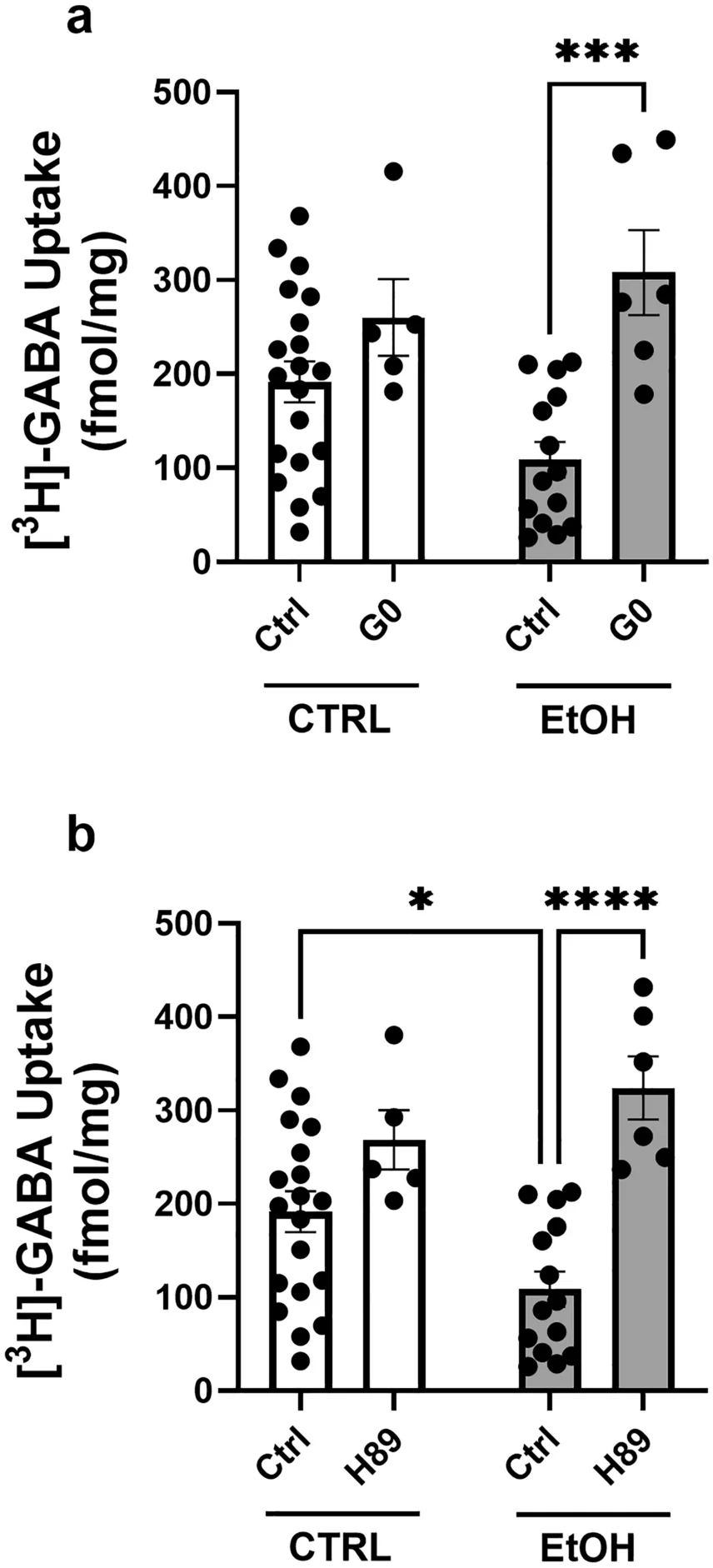
Inhibition of PKA and PKC reverses EtOH-induced reduction in GABA uptake in E11 chicken retinas. **A** GABA uptake was reduced following 30-min treatment with 0.1% ethanol (EtOH), however, this comparison did not reach statistical significance (*p* = 0.0511). Treatment with the PKC inhibitor Gö6983 (100 nM) increased uptake (*p* = 0.0002 vs. EtOH) while it did not induce any effect on control group (p = 0.4523 vs. CTRL). **B** Similarly, the PKA inhibitor H89 (10 µM) reversed the EtOH-induced reduction in GABA uptake (p < 0.0001 vs. EtOH), whereas H89 alone did not significantly affect GABA uptake (p = 0.2852 vs. CTRL). Data are presented as mean ± SEM (n = 5–20). Statistical analysis was performed using Welch’s one-way ANOVA followed by Šidák’s post hoc test. Different colors for individual dots correspond to different experiment batches. *p < 0.05, *** p < 0.001 and **** p < 0.0001

Since our data suggest that EtOH-decreased GABA uptake does not involve modulation of GAT levels, we asked whether PKA- and PKC-dependent signaling pathways contribute to this effect, as both kinases are central to control GAT activity. In this context, we tested whether pharmacological blockade of each enzyme could reverse EtOH-induced effects (Fig. 9 A, B). To do so, E11 retinal explants were treated for 30 min with 0.1% EtOH alone or in combination with either 10 mM H-89 (PKA inhibitor), or 100 nM Gö6983 (PKC inhibitor). Uptake of [^3^H]-GABA was then quantified and compared across experimental groups. For PKC analysis, two-way ANOVA test did not point to significance with EtOH treatment (F_1,41_ = 0.2999; p = 0.5869) but detected a significant statistical difference for treatment with G0 (F_1, 41_ = 17.62; p = 0.0001) and interaction between drugs (F_1, 41_ = 4.172; p = 0.0476). As shown in Fig. 9A, GABA uptake was reduced following 30-min treatment with 0.1% EtOH, however, this comparison did not reach statistical significance (p = 0.0511 vs. EtOH), confirming previous observations. Notably, Gö6983-treated retinas did not differ from control (p = 0.4523 vs. CTRL). However, when compared to EtOH, G0 significantly increased GABA uptake levels (p = 0.0002 vs. G0 + EtOH), indicating that its primary effect is to counteract EtOH’s inhibition rather than to elevate uptake beyond baseline. Similarly, for PKA analysis, the two-way ANOVA test did not indicate a statistical difference with EtOH treatment (F_1,41_ = 0.2054; p = 0.6528) but found a significant statistical difference for treatment with H89 (F_1, 41_ = 23.72; p < 0.0001), with interaction between drugs (F_1, 41_ = 5.314; p = 0.0263). While EtOH decreased GABA uptake (p = 0.0335 vs. CTRL), the PKA inhibitor H-89 did not affect GABA uptake by itself (p = 0.285 vs. CTRL) but increased GABA uptake relative to EtOH alone (p < 0.0001 vs. H89 + EtOH) (Fig. 9B**)**, suggesting that the observed increase reflects reversal of EtOH’s effect rather than enhancement above physiological levels.

These results provide evidence that the inhibitory effect of EtOH on GABA uptake is mediated, at least in part, by signaling pathways dependent on PKA and PKC. Pharmacological inhibition of either kinase is sufficient to normalize transporter function, supporting the idea that EtOH acts through these pathways to suppress GABA uptake and that blocking them restores uptake to control levels without over-shooting baseline.

## Discussion

### Acute EtOH Affects GABA Uptake in a Dose-Dependent Manner

 Several drugs of abuse have been investigated using the chicken retina model exploring its unique neuro-glial circuit, such as caffeine [43], nicotine [38], EtOH [22], opiates [44] and cannabinoids [45, 46], among others [47]. Here, we demonstrate that a single exposure to 0.1% EtOH at the onset of synaptogenesis is probably enough to modify GABA homeostasis. A 30-min exposure was used to assess acute effects, consistent with previous studies in the chicken retina employing similar incubation times [22, 38, 45].

The presence of functional GABA uptake at E11 is consistent with the early establishment of GABAergic circuitry during retinal development [36, 48]. Consistent with these trends, [^3^H]-GABA uptake assays revealed a functional transport system at E11 and a ~50% increase by E16, reflecting progressive maturation of GABA transport during synaptogenesis. Importantly, previous studies from our group demonstrated that [^3^H]-GABA uptake is completely abolished when extracellular Na⁺ ions are replaced by Tris and markedly reduced at 4 °C, providing strong evidence that the measured signal reflects a specific sodium-dependent, transporter-mediated process requiring metabolic activity rather than nonspecific radiotracer incorporation [17]. Moreover, a single dose of 0.1% EtOH exposure at E11 suggested a reduction in GABA uptake without alteration in tissue viability or total GAT-1 levels. These effects disappear after EtOH washout, highlighting a reversibility mechanism. Importantly, the recovery of GABA uptake following washout indicates that the effect depends on the continuous presence of EtOH and does not reflect long-lasting or irreversible alterations in transporter function. The rapid recovery of the system is consistent with a dynamic modulation of transporter activity, potentially involving phosphorylation-dependent processes. However, we acknowledge that this assay alone does not resolve the precise temporal dynamics of EtOH action. A detailed time-course study, including pretreatment intervals and co-application protocols, would be necessary to distinguish between acute interference and downstream regulatory effects.

Although GAT-1 activity is relevant in E11, other high-affinity GABA transporters may contribute to total uptake at both stages [49]. Furthermore, the significant reduction in [3H]-GABA uptake produced by NO-711 here supports the participation of a GAT-1-mediated component in this process. Such early functionality aligns with the broader developmental roles of GABA in regulating proliferation, neuronal survival, differentiation and synapse formation [50, 51]. Alternatively, additional GABA derived from putrescine via alternative metabolic routes also supports early inhibitory tone in mammals, a mechanism that may also operate in the chicken retina at E11 when endogenous synthesis is still increasing [52].

EtOH, as a lipophilic molecule, might cross both the blood–brain and the placental barrier, due to its solubility, causing a wide range of toxicity [53]. Moreover, as stated by the WHO, no level of alcohol consumption is safe. In addition, doses that seem minimal already have an impact on neurodevelopment [54]. The dose–response curve ranging from 0.1% to 0.5% suggests a decrease in GABA uptake at 0.1%, with no incremental inhibition at higher doses. Comparable low-dose sensitivity has been documented in cortical cultures, where sub-millimolar EtOH alters synaptic viability and neurotransmitter release [55]. The 0.1% EtOH used here corresponds to ~ 0.1 g dL⁻1 (21.71 mM), an equivalent blood-alcohol level achieved in humans after a moderate-to-high consumption (four standard drinks in two hours) and widely employed in FASD research to model episodic maternal drinking. GABA transporters, particularly GAT-1, constitute early molecular targets of EtOH [56]; their modulation may influence the establishment of neural networks during critical periods of circuit assembly.

Future studies should determine whether this inhibition involves direct transporter–EtOH interactions, EtOH-induced shifts in membrane potential, or additional signaling cascades beyond PKA/PKC pathways, thereby clarifying how transient maternal alcohol exposure can leave lasting effects on inhibitory circuitry.

### Inhibition of GAT‑1 Activity by EtOH is not Linked to a Downregulation of GAT-1

GAT-1 function is known to be regulated by phosphorylation-dependent mechanisms that affect transporter activity and membrane trafficking. Our previous work showed that GAT-1 can be modulated by drugs such as caffeine and cocaine [17, 39, 57]. In this sense, our results indicate that the suggestive acute reduction in GABA uptake induced by EtOH is not accompanied by changes in the total expression of GAT-1 protein within the 30-min treatment window. Hence, the inhibition we observe is unlikely to originate from transcriptional or translational down-regulation of the transporter and instead points to fast functional modulation of GAT-1. Similar discrepancies between function and abundance have been reported in other brain regions after chronic EtOH exposure, where altered uptake kinetics were detected despite unchanged GAT-1 levels [58]. In E11 chicken retinae, a 0.1% EtOH pulse lowered basal [3H]-GABA uptake by ~43.9%, indicating that EtOH directly impairs transporter function. Application of the selective GAT-1 inhibitor NO-711 reduced uptake even further (~ 53.5% versus baseline), confirming its potency. Addition of EtOH with NO-711 produced a ~67% decrease of [^3^H]-GABA uptake relative to control values, with a non-additive interaction between EtOH and pharmacological blockade at this early developmental stage. Furthermore, the persistence of a certain level of uptake even with pharmacologically blocked GAT-1 is due to the presence and functionality of GAT-3, which performs a small portion of uptake and release in glial cells of the retinal tissue [29]. To our knowledge, this is the first study to examine low-dose (0.1% v/v) EtOH effects on GABA uptake in E11 retina with NO-711, highlighting the transporter’s vulnerability at the onset of synaptogenesis. Although GAT-1 is a major contributor, other high-affinity transporters probably participate, as developmental surveys have shown multiple GAT isoforms in the retina [49]. Tyrosine phosphorylation of GAT-1 is known to modulate its transport capacity [59]. The absence of a measurable change in GAT-1 protein suggests that EtOH acts through post-translational or signaling-dependent mechanisms.

### Reversible EtOH‑Induced Inhibition of GABA Uptake Occurs Without Cytotoxicity in E11 Retinas

The increase in LDH release is often correlated with the severity of EtOH exposure and is a reliable indicator of retinal damage [60]. During retinal development in the chick, a wave of programmed cell death unfolds between E10 and E14, peaking at E11, particularly within the inner nuclear layer [9, 61]. Since E11 is a period of intense cell death, one question that remains to be answered is whether cell death is being potentiated by EtOH. Previous work has demonstrated that an acute exposure to 1% EtOH in human retinal organoids led to a significant increase in apoptosis and cell cycle block, reflecting cell death and significant alterations in neuronal differentiation [62]. As LDH release was not further increased upon acute 0.1% EtOH challenge, this supports the interpretation that the observed reduction in GABA uptake reflects a transient functional modulation of GABA transporters rather than cell damage. A comparable phenomenon was reported by [63], who used the same LDH assay in murine retinal explants and showed that activation of the lactate-sensitive receptor HCA1R with 3.5 mM 3,5-DHBA preserved neuronal viability without elevating LDH. We reinforce that membrane integrity is maintained and that EtOH-induced interference (0.1%) with the GABAergic system in E11 chicken retina constitutes a physiological adjustment, not a cytotoxic event. It is important to note that, as the experiment was performed ex vivo, a basal level of LDH release is expected due to the intrinsic stress of tissue dissection and handling. Nonetheless, the absence of increased LDH release in the EtOH group supports the conclusion that the functional modulation of GABA uptake observed previously occurs in the absence of extensive cell damage. These findings strengthen the interpretation that the apparent effect in GABA uptake induced by acute EtOH exposure reflects a physiological regulatory mechanism rather than a pathological response associated with cell death. Following that, we hypothesized that, functionally, if we remove EtOH from the challenge after a previous exposure, we would be able to have the activity return, reinforcing that we are not experiencing death. The ability of developmental systems to recover from EtOH exposure may be linked to the timing and dosage of exposure. Studies in mice have shown that the timing of EtOH exposure significantly influences the severity and persistence of behavioral deficits, suggesting that early intervention or removal of EtOH can mitigate long-term effects [64]. In *C. elegans*, developmental delays were observed during and immediately after EtOH exposure (10%), but the effects were dynamic and not uniform, indicating that some recovery is possible after the removal of EtOH in these terms [65]. Similarly, research using zebrafish models has shown that short-term exposure to EtOH (low doses at 24-h post-fertilization and up to 2 h of exposure) might lead to developmental disruptions, but these effects may not be permanent [66]. For instance, zebrafish embryos exposed to EtOH exhibited behavioral anomalies, yet these were not always sustained into adulthood, suggesting a potential for recovery [67]. While the potential for recovery from EtOH-induced disruptions is promising, it is important to consider that the reversibility may vary across different species and developmental stages. These findings contribute novel functional evidence that short-term exposure to EtOH during critical developmental windows may produce transient, rather than sustained, disruption of GABA transporter function. To our knowledge, this is the first report to evaluate the reversibility of EtOH-induced inhibition of GABA uptake in the embryonic chicken retina, particularly at low EtOH concentrations.

### EtOH Does not Modify GluN2B Phosphorylation but Promotes GABA Release in E11 Retinas

NMDA receptor signaling—particularly involving the GluN2B subunit—has been identified as a potential mediator of EtOH’s effects in the developing nervous system [68]. Previous studies from our group have shown that pharmacological agents such as caffeine might modulate GluN2B-dependent signaling in the embryonic retina, which is blocked by Ifenprodil [18]. EtOH exposure might also modulate NMDA receptor function, potentially through phosphorylation of GluN2B subunits, leading to altered glutamatergic neurotransmission [69]. Although GluN2B is a known molecular target of EtOH in several brain regions and developmental models [8], our findings indicate that this specific signaling pathway is probably not involved during short-term exposure at this stage of retinal development (E11). Our results demonstrated that EtOH exposure significantly increased GABA release compared to control. Specifically, the amount of [^3^H]-GABA released during the 5-min pulse more than doubled in the EtOH-treated group relative to the vehicle-treated group (Fig. 8). To test whether this effect involved GluN2B-containing NMDA receptors, we included Ifenprodil, an allosteric inhibitor of GluN1-GluN2B subunits, during the EtOH pulse. Co-application of Ifenprodil with EtOH did not significantly reduce GABA release compared to EtOH alone.

The GABAergic system plays a crucial role in retinal development, and EtOH exposure can modulate GABA release through multiple mechanisms [70]. Activation of group III metabotropic glutamate receptors by EtOH decreases GABA immunoreactivity and alter glutamate release in the chicken retina, suggesting a complex interaction between glutamatergic and GABAergic systems [71]. Additionally, EtOH exposure can potentiate the depolarizing action of GABA in GABAergic interneurons, leading to aberrant migration of these cells in the embryonic cortex [72, 73]. Moreover, EtOH exposure has been shown to disrupt calcium signaling in the developing retina, which is critical for GABA release [74]. Several molecular targets may mediate the EtOH-induced increase in GABA release in the E11 chicken retina, since this effect was not blocked by Ifenprodil, whose pharmacological actions at 10 μM include near-maximal inhibition of GluN2B-containing NMDA receptors (IC₅₀ = 0.34 μM) [75]. The complete absence of an Ifenprodil effect suggests that GluN2B-containing NMDA receptors are not critically involved in this process. This negative result, while not definitive, did not warrant further investment in more selective GluN2B antagonists.

### Inhibition of PKA and PKC Reverses EtOH-Induced Reduction in GABA Uptake

Protein kinase A (PKA) and C (PKC) are essential serine/threonine kinases involved in diverse cellular functions [76, 77]. These kinases exert contrasting effects on GABAergic signaling, vital for inhibitory neurotransmission in the developing retina. PKA and PKC influence the trafficking, surface expression, and functionality of GABAA receptors, which are critical for GABA uptake and synaptic inhibition [55, 78–80]. After observing a suggestive reduction in GABA uptake following acute EtOH exposure in the developing retina without altering GAT-1 expression or inducing cytotoxicity, and that this effect is reversible upon EtOH removal, we next sought to investigate the intracellular signaling pathways potentially involved in this modulation.

This uptake reduction is sensitive to pharmacological inhibition of GAT-1 activity and is abolished by pharmacological inhibition of PKA or PKC pathway, suggesting the involvement of kinase-dependent signaling in transporter regulation. Previous studies from our group have consistently [20] demonstrated the involvement of kinase-dependent signaling in the regulation of GABA transport in the chicken retina: Caffeine-induced reduction in GABA uptake in the chicken retina is mediated via PKA-dependent mechanisms [17], while caffeine also potentiates GAT-1-mediated D-aspartate-induced GABA release via adenosine A1 receptor inhibition and PKA activation [18]. Furthermore, pharmacological inhibition of PKC attenuates the nicotine-induced modulation of GABA uptake [38], reinforcing the role of intracellular kinase signaling in the drug-induced regulation of GABA transporter function in the chicken retina. Additionally, literature supports that EtOH can influence both PKA and PKC signaling pathways in neuronal systems [55, 81]. The activation of PKA by EtOH significantly enhances the surface expression of GABAA α1 subunits in cerebral cortical neurons, correlating with increased zolpidem potentiation of GABA responses, thus suggesting a facilitative role of PKA in GABAA receptor functionality [79]; concurrently, PKA activation also influences extra synaptic GABAA α4δ receptors, promoting tonic inhibition and potentially contributing to EtOH's neuroprotective properties under very specific conditions [80]. On the other hand, EtOH exposure activates PKC, specifically the PKCγ isoform, resulting in the internalization of GABAA α1 subunits, thereby diminishing their surface expression and impairing GABAergic inhibition, which may contribute to the hyperexcitability observed in the developing retina [82, 83]. The interplay of PKA and PKC within GABAergic signaling is not autonomous but rather interconnected with various signaling cascades; for instance, EtOH-initiated PKA activation is regulated by GABAB receptor activity, influencing intracellular cAMP levels and CREB phosphorylation [73, 84], while PKC activation through EtOH is affected by glycine receptor modulation, potentially leading to the inhibition of glycine-activated currents in retinal neurons [85]. To our knowledge, this is the first study to provide evidence supporting the involvement of kinase-dependent intracellular signaling in the modulation of GABA transport by acute EtOH in the developing chicken retina. Although additional mechanisms cannot be excluded, these findings identify PKA- and PKC-dependent signaling as a plausible component of the acute response and provide a basis for future mechanistic studies.

### Study Limitations

While some experimental groups in this study had unbalanced or modest sample sizes, we believe this does not reduce the biological consistency or relevance of our findings. Across different assays, consistent patterns were observed in independently repeated experiments, supporting a coherent biological effect. In specific cases such as Western blot analyses, statistical testing was conducted even when group sizes were small (n = 3), and although no significant differences were found, these results were interpreted as preliminary indications of the absence of detectable changes in marker expression, rather than definitive conclusions. All experiments were performed under standardized conditions, and positive controls such as Triton X-100 were used to confirm assay sensitivity, while excluded from inferential statistics when not appropriate. While we acknowledge the limitations of some comparisons in terms of statistical power, we prioritized internal consistency, biological plausibility, and reproducibility across assays. Taken together, the data present a robust and biologically grounded model of EtOH acute effects on GABAergic transport during retinal development, contributing valuable insights and guiding future confirmatory studies.

## Conclusion

This study identifies that acute EtOH exposure (0.1% at an early stage of synapse development, at E11) suggested a reduction in GABA uptake in the embryonic chicken retina without changing total GAT-1 abundance. The effect is reversible and is associated with the involvement of PKA and PKC signaling pathways, suggesting that a post-translational regulation may contribute to reduced transporter function rather than expression. Because GABA transport is already active at E11 and increases by E16, the acute EtOH-induced modulation of GABA clearance observed in the present study may be relevant to retinal circuit establishment. Co-application with NO-711 further lowers uptake, consistent with GAT-1 representing a major contributor to the EtOH-sensitive component of GABA uptake, while not excluding roles for other high-affinity GABA transporters. The combination of reversibility, kinase dependence, and unchanged protein levels is consistent with dynamic control of transporter activity through mechanisms that may involve phosphorylation and/or membrane trafficking as a plausible mechanism. Future studies should unravel the molecular steps linking acute EtOH to GAT-1 control (e.g., signaling, surface trafficking kinetics, transporter interactors) and test whether similar rules apply at other developmental stages and for other GABA transporter isoforms.

## Supplementary Information

Below is the link to the electronic supplementary material.

Supplementary file1 (XLSX 210 kb)

## Data Availability

No datasets were generated or analysed during the current study.

## Abbreviations

ANOVA: Analysis of variance
ATP: Adenosine triphosphate
BSA: Bovine serum albumin
CMF: Calcium- and magnesium-free (solution)
CNS: Central nervous system
DMEM: Dulbecco’s Modified Eagle Medium
E11: Embryonic day 11
E16: Embryonic day 16
E6: Embryonic day 6
E8: Embryonic day 8
ECL: Enhanced chemiluminescence
EMCCD: Electron multiplying charge-coupled device
EtOH: Ethanol
F12: Ham’s F-12 nutrient mixture
FASD: Fetal Alcohol Spectrum Disorders
Fura‑2/AM: Calcium indicator dye Fura‑2 acetoxymethyl ester
GABA: Gamma-aminobutyric acid
GABA-A: Gamma-aminobutyric acid receptor type A
GAT: Gamma-aminobutyric acid transporter
GAT-1: Gamma-aminobutyric acid transporter 1
GluN1: NMDA receptor subunit 1
GluN2B: NMDA receptor subunit 2B
Gö6983: Protein kinase C inhibitor
H-89: Protein kinase A inhibitor
HBSS: Hank’s Balanced Salt Solution
KCl: Potassium chloride
LDH: Lactate dehydrogenase
mRNA: Messenger ribonucleic acid
NMDA: N-methyl-D-aspartate
NO-711: Selective GABA transporter 1 inhibitor
NR2B: NMDA receptor subunit 2B
PKA: Protein kinase A
PKC: Protein kinase C
PVDF: Polyvinylidene difluoride
RIPA: Radioimmunoprecipitation assay buffer
SDS: Sodium dodecyl sulfate
SDS-PAGE: Sodium dodecyl sulfate polyacrylamide gel electrophoresis
